# *In vivo* characterization of early-stage radiation skin injury in a mouse model by two-photon microscopy

**DOI:** 10.1038/srep19216

**Published:** 2016-01-12

**Authors:** Won Hyuk Jang, Sehwan Shim, Taejun Wang, Yeoreum Yoon, Won-Suk Jang, Jae Kyung Myung, Sunhoo Park, Ki Hean Kim

**Affiliations:** 1 Divison of Integrative Biosciences & Biotechnology, Pohang University of Science and Technology, 77 Cheongam-Ro, Nam-gu, Pohang, Gyeongbuk 37673, Rep. of Korea; 2 National Radiation Emergency Medical Centre, Korea Cancer Centre Hospital, Korea Institute of Radiological & Medical Sciences (KIRAMS), 75 Nowon-ro, Nowon-gu, Seoul 01812, Rep. of Korea; 3 Department of Mechanical Engineering, Pohang University of Science and Technology, 77 Cheongam-Ro, Nam-gu, Pohang, Gyeongbuk 37673, Rep. of Korea; 4 Laboratory of Experimental Pathology, Korea Cancer Centre Hospital, Korea Institute of Radiological & Medical Sciences (KIRAMS), 75 Nowon-ro, Nowon-gu, Seoul 01812, Rep. of Korea; 5 Department of Pathology, Korea Cancer Centre Hospital, Korea Institute of Radiological & Medical Sciences (KIRAMS), 75 Nowon-ro, Nowon-gu, Seoul 01812, Rep. of Korea

## Abstract

Ionizing radiation (IR) injury is tissue damage caused by high energy electromagnetic waves such as X-ray and gamma ray. Diagnosis and treatment of IR injury are difficult due to its characteristics of clinically latent post-irradiation periods and the following successive and unpredictable inflammatory bursts. Skin is one of the many sensitive organs to IR and bears local injury upon exposure. Early-stage diagnosis of IR skin injury is essential in order to maximize treatment efficiency and to prevent the aggravation of IR injury. In this study, early-stage changes of the IR injured skin at the cellular level were characterized in an *in vivo* mouse model by two-photon microscopy (TPM). Various IR doses were applied to the mouse hind limbs and the injured skin regions were imaged daily for 6 days after IR irradiation. Changes in the morphology and distribution of the epidermal cells and damage of the sebaceous glands were observed before clinical symptoms. These results showed that TPM is sensitive to early-stage changes of IR skin injury and may be useful for its diagnosis.

Ionizing radiation (IR), including X-ray and gamma ray, is widely used in both medical and industrial fields. Either accidental or therapeutic exposure to high dose of IR can induce severe damage in living tissues. IR induces irreversible double-strand breaks in the deoxyribonucleic acid (DNA) of cells and various other harmful effects by generating free radicals. Organs having highly proliferative and sufficiently oxygenated cells are the most sensitive to IR, including the bone marrow, reproductive and gastrointestinal systems, vascular systems, skin, muscle, and brain^1^,^2^,^3^. Localized radiation injuries are frequent, and the skin is the first organ exposed to IR. Skin injury by IR involves immediate damage to basal keratinocytes and hair follicle stem cells, followed by a burst of free radicals, irreversible double-strand DNA breaks, and inflammation. IR-induced skin injury can have different grades of severity ranging from erythema, dry and moist desquamation, to ulceration and necrosis^4^. Radiation skin injuries have specific characteristics of the post-irradiation delay in the onset of clinical changes and the successive and unpredictable inflammatory bursts. The mechanism about the cascade of inflammatory mediators and continued activation of immune cells is not well understood.

Treatment of radiation skin injury is a challenge unresolved by the therapeutic approach for other skin injuries such as thermal and chemical burns, due to its differences in terms of patho-physiological mechanisms, clinical aspects, and progression^5^,^6^. In case of severe radiation skin injuries, surgeons may face technical difficulty in treatment due to the occurrence of successive and unpredictable inflammatory waves with progressive extension of necrosis in both superficial and deep layers^7^. If these successive and unpredictable inflammatory waves impair the previous therapeutic approach, the only possible approach is additional surgical excision. Moreover, such successive inflammatory waves are associated with uncontrollable pain and often show resistance to analgesics. Therefore, early and accurate diagnosis is essential to minimize the expansion of injured area and to increase the treatment efficacy.

Diagnostic methods to detect the radiation injury were based on visible clinical signs, numerical dosimetry reconstruction, cytogenetic analysis, and other physical parameters. Diagnosis based on visible clinical sign is easy and convenient than other methods, but have limitations due to the clinically latent period. Numerical dosimetry reconstruction analyses the absorbed radiation dose and damage based on a mathematical model^8^,^9^. However, such numerical dosimetry reconstruction is indirect and often not applicable in practice because the simulation requires a detailed knowledge of the scenario such as the exposure type, distance between the radioactive source and lesion, and duration of the exposure. Dose physical reconstruction based on *ex vivo* electron paramagnetic resonance (EPR) measurements have been used for evaluation, but this method is invasive because of the need to extract bone samples^10^,^11^. In contrast to EPR measurement, bio-dosimetric techniques such as fluorescence *in situ* hybridization (FISH), premature chromosome condensation (PCC) assay and hypoxanthine-guanine phosphoribosyl transferase (HPRT), use peripheral blood sample for analysis. However, these techniques are not effective for diagnosis of localized IR-injury. For high-throughput gene expression methods, serial analysis of gene expression (SAGE), oligonucleotide arrays, and cDNA arrays are used^12^,^13^,^14^. Recently, assays to detect the increase of several serum protein levels and skin injury protein biomarkers have been proposed^15^,^16^.

Optical imaging techniques have been applied as the diagnostic method to overcome the limitations of previous methods. These optical techniques can non-invasively detect various areas of IR induced skin injuries. Therefore, these techniques may allow mapping of the radiation injured area. Laser speckle imaging (LSI) measures laser speckle patterns generated by light scattering in the skin and can detect the perfusion changes. LSI has been applied to diagnose the of radiation skin injury using a pig model, which showed the ability to distinguish the healthy and irradiated skin areas during the clinically latent period^17^. Hyperspectral imaging (HSI) is a wide-field imaging technique which measures spectral changes of reflected light in broad ranges. HSI has been used for detection of acute changes in oxygenation and perfusion following irradiation^18^. Based on the level of oxy-haemoglobin (Hb-oxy) and deoxy-haemoglobin (Hb-deoxy), the tissue oxygenation level remained above baseline depicting the area of irradiation. Diffuse optical spectroscopy (DOS), which is a similar technique to HSI, allows assessment of functional changes in tissues based on haemoglobin content, oxygen saturation, and tissue scattering power. DOS has been applied to differentiate the skin toxicities induced by IR injury^19^. LSI, HIS, and DOS detect physiological or functional changes due to vascular damage and do not provide cellular information. Reflectance confocal microscopy (RCM) is a non-invasive high-resolution 3D imaging technique based on light reflection and can image tissues at the cellular resolution. RCM was applied to acute radiation dermatitis (ARD) patients and visualized various histopathological changes at the cellular level including spongiosis, exocytosis, and dense infiltration of inflammatory cells before the clinical onset^20^.

In this study, we used two-photon microscopy (TPM) to characterize early-stage changes of radiation skin injury at the cellular level in a mouse model *in vivo*. TPM is another high-resolution 3D imaging technique based on nonlinear two-photon excitation of fluorophores, which can provides molecular and cellular information of the tissues^21^,^22^,^23^. TPM can image 3D cellular structures in the skin based on either autofluorescence or extrinsic fluorescent labelling. TPM has advantages of higher imaging depths, minimal photo-damage compared to confocal microscopy. TPM has been used in various skin studies including transdermal transport pathways^24^, cutaneous immune response^25^, skin melanoma or cancer detection^26^,^27^. TPM is expected to detect cellular changes in the skin, similarly to RCM, but with autofluorescence contrast.

TPM was applied for characterizing the early stage cellular changes of IR injured skin in a mouse model. Various IR doses were irradiated onto the mouse hind limb, and changes from day 1 to day 6 after irradiation were monitored by longitudinal two-photon (TP) imaging. Cellular changes in the skin epidermis and sebaceous gland were characterized. Results of TP imaging were compared with white light images and histology results.

## Results

### TP images of the skin epidermis in the early stage of IR injury

**Figure 1:**
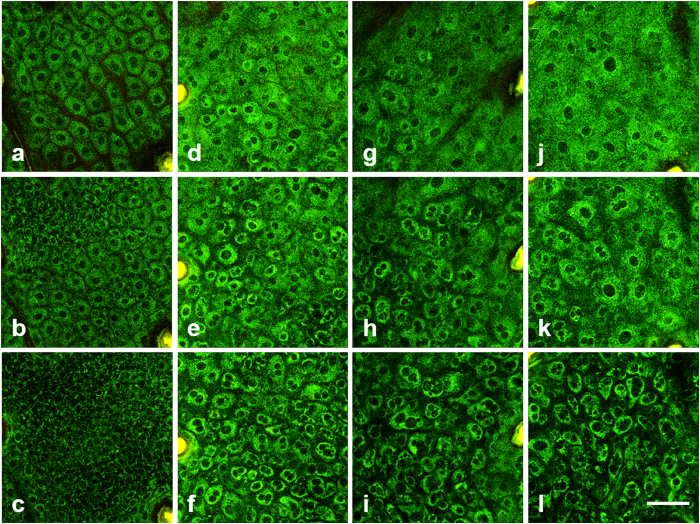
TP 3D images of the skin epidermis on day 6 after irradiation with various IR doses. (**a–l**): TP images of the stratum granulosum, stratum spinosum, and stratum basale with 0 Gy (control), 20 Gy, 30 Gy, and 40 Gy dose, respectively. Scale bar indicates 50 μm.

In order to detect cellular changes in the epidermis in the early stage, TP images of the irradiated skin on day 6 after IR irradiation are shown in Fig. 1 (see Supplementary Information for related videos). TP images of the irradiated skin, which were exposed by various IR doses from 0 Gy (control), 20 Gy, 30 Gy, 40 Gy, are shown in different columns from left to right. Several layers in the epidermis: stratum granulosum, stratum spinosum, and stratum basale, are presented in different rows from top to bottom. TP images of the control skin showed normal cell morphology and distribution: uniform cell size in each layer and densely distributed with thin and uniform intercellular spaces. Cells are relatively large and flat in the superficial stratum granulosum, and become smaller in the deeper layers. Therefore, the cell density, which could be defined as the number of cells per unit area, increases with depth. TP images of the irradiated skin with various IR doses show different cell morphologies and distributions on day 6 after irradiation. Cells in each layer are irregular in both size and shape. In the stratum granulosum, cell boundaries are less clear compared to those in the control skin. Cell shapes are irregular, and some cells have multiple nuclei. In the stratum spinosum and stratum basale, cell shapes are irregular and enlarged, and more cells with multiple nuclei are found. The intercellular spaces between cells have also increased. With enlargement of individual cells and increased intercellular spaces, the cell density decreases compared to that of the control skin. These changes are found in all the irradiated skin images on day 6 after exposure and are more significant with higher IR doses.

### Longitudinal TP images of the stratum basale in the early stage of IR injury

**Figure 2:**
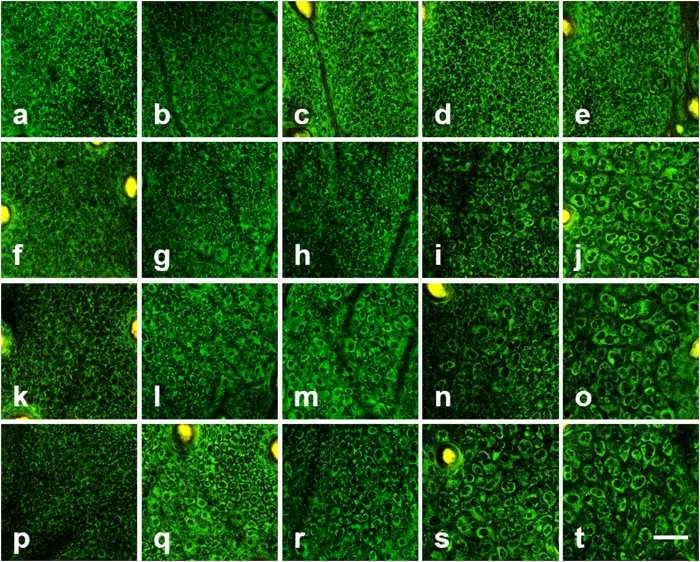
Longitudinal TP images of the stratum basale in the early stages of IR injury. (**a–t**): TP images on day 1 and 3–6 after irradiation with 0 Gy (control), 20 Gy, 30 Gy, and 40 Gy doses, respectively. Scale bar indicates 50 μm.

In order to characterize the progression of early-stage cellular changes in the epidermis, longitudinal TP images of the skin in the stratum basale, irradiated with different IR doses of 0 Gy (control), 20 Gy, 30 Gy, and 40 Gy, are shown in Fig. 2(a–t), respectively. TP images on day 1 and 3–6 after irradiation are presented in different columns from left to right. TP images of the control skin showed densely-packed small-sized cells in the stratum basale. On day 1 after irradiation, all groups show no definite changes from that of the control skin: small and uniform sized cells are densely packed. On day 3–6 after irradiation, some cellular changes are detected in the irradiated skin samples. TP images of the stratum basale in 30 Gy and 40 Gy showed changes in cellular morphology and distribution on day 3 or 4 after irradiation. The ones with 20 Gy irradiation showed such changes at a later time: on day 4 or 5 after irradiation. On day 5 after irradiation, TP images of the stratum basale with 30 Gy and 40 Gy irradiation showed increased intercellular spaces. Similar phenomena appeared in TPM images with 20 Gy irradiation on day 6 after irradiation. On day 6 after irradiation, TP images of all the irradiated skin samples showed significant changes in cell morphology and distribution compared to those of the control skin.

### Longitudinal TP images of the sebaceous gland in the early stage of IR injury

**Figure 3:**
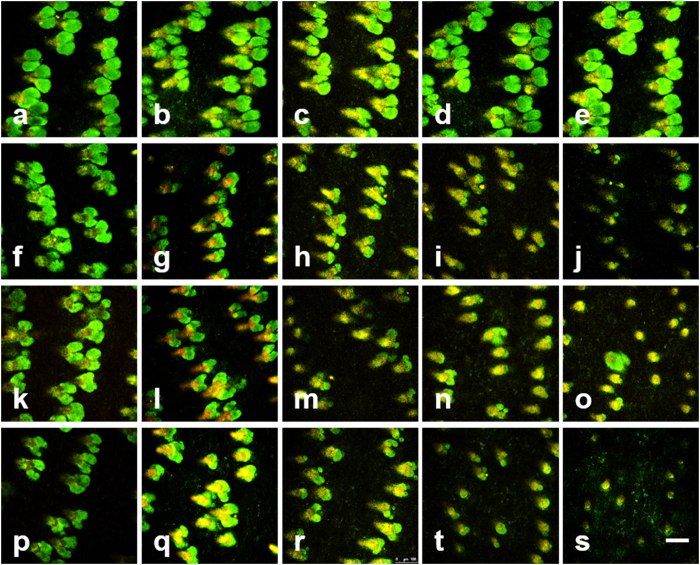
Longitudinal TP images of the sebaceous gland in the early stages of IR injury. (**a–t**): TP images on day 1 and 3–6 after irradiation with 0 Gy (control), 20 Gy, 30 Gy, and 40 Gy doses, respectively. Scale bar indicates 100 μm.

TP images of the sebaceous gland in the irradiated skin from day 1 and 3–6 after irradiation are shown in Fig. 3. Longitudinal TP images of the sebaceous glands irradiated with different IR doses of 0 Gy (control), 20 Gy, 30 Gy, and 40 Gy are shown in different rows from top to bottom. TP images on day 1 and day 3–6 after irradiation are shown in different columns from left to right. TP images of the control skin showed multiple sebaceous glands attached to individual hair follicles. These sebaceous glands appear in TP images due to autofluorescence from both cells and molecules in the glands. On day 1, TP images of all the irradiated skin cases showed that the glands are still normal in both size and shape as the control ones. TP image of the sebaceous glands with 40 Gy shows changes on day 4 after irradiation: glands become rounder and smaller compared to the normal ones. This might be due to cell death in the sebaceous glands. These changes progress onto day 6 after irradiation: some glands became even smaller and some disappeared. TP images of the sebaceous gland with lower IR doses than 40 Gy showed similar changes, and these changes became obvious in all the irradiated groups on day 6 after irradiation.

### Longitudinal white light images of the mouse skin in the early and middle stage of IR injury

**Figure 4:**
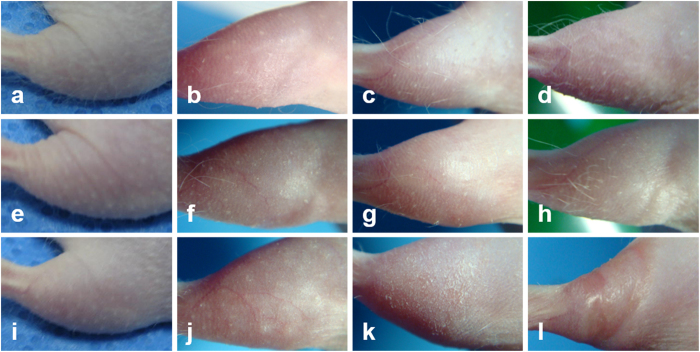
Longitudinal white light images of the irradiated mouse hind limbs. (**a–l**): white light images on day 2, 6, 9, and 13 after irradiation with 0 Gy (control), 20 Gy, and 40 Gy doses, respectively.

In order to observe visual changes in the skin after irradiation, white light images were taken longitudinally along with TP imaging and they are presented in Fig. 4. These white light images were taken of mice which had been irradiated in the same condition as the ones imaged by TPM. Longitudinal white light images of the mouse right hind limbs, irradiated with various IR doses of 0 Gy (control), 20 Gy, and 40 Gy, are shown in Fig. 4(a–l), respectively. The images on day 2, 6, 9, and 13 after irradiation are shown from left to right. White light images of the control mouse skin show normal skin structure of the hairless mouse: rough skin surface and sparse hair distribution. Images of the irradiated skins show structural changes: (1) on day 2 after irradiation, the irradiated skins with both 20 Gy and 40 Gy show no sign of changes, (2) on day 6 after irradiation, the ones with 20 and 40 Gy show partial and complete hair loss respectively, (3) on day 9 after irradiation, the one with 40 Gy shows dry desquamation and the one with 20 Gy shows no further change, (4) on day 12 after irradiation, the one with 40 Gy shows necrosis and the one with 20 Gy showed hair regrowth. TP imaging was conducted until day 6 after irradiation, and the white light images show not many changes other than hair loss by that time.

### Histological images of the mouse skin in the early stage of IR injury

**Figure 5:**
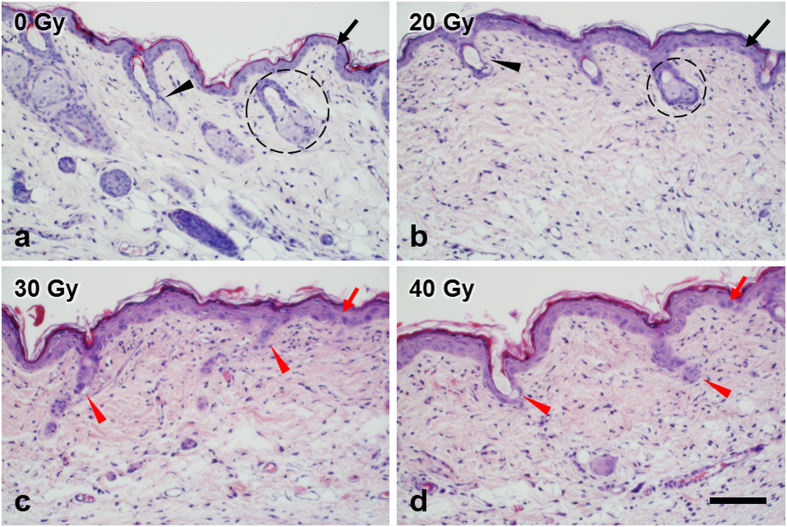
H&E stained histological images of the mouse hind limb skin on day 6 after irradiation. (**a–d**): skin images with 0 Gy (control), 20 Gy, 30 Gy, and 40 Gy dose irradiation, respectively. In figure; epithelium (black arrow), hair follicle (black arrowhead), sebaceous gland (dashed circle), thickened epithelium with irregular cell arrangement (red arrow), loss of hair follicles and sebaceous glands (red arrow head). Scale bar indicates 100 μm.

To confirm the TPM study results, the mouse skin samples were processed for histological evaluation. Histological images of the mouse skin on day 6 after irradiation with various doses of 0 Gy (control), 20 Gy, 30 Gy, and 40 Gy are shown in Fig. 5. These histological images show microscopic changes in both the epidermis and dermis, although only the epidermis and superficial dermis were imaged by TPM in this study. Various features in the skin and cellular changes due to IR injury were labelled with different markers. Histological image of the control skin showed no alteration of normal structures of the epidermis and dermis: thin epithelium (black arrow), regularly distributed hair follicle (black arrowhead) and sebaceous gland (dashed circle), and regular cell distribution in the dermis. Histological images of the irradiated skin samples showed several changes compared to the one of the control skin. The one with 20 Gy irradiation showed not many changes from the control: normal epithelium thickness, normal or a slightly smaller sebaceous glands, and regular cell distribution in the dermis. The ones with 30 Gy and 40 Gy irradiation showed some prominent changes: thickened epithelium with irregular cell arrangement (red arrow), loss of hair follicles and sebaceous glands (red arrowhead). These histological images showed consistent results found by the *in vivo* TPM study.

### Quantitative analysis of cellular changes in TP images in the early stage of IR injury

**Figure 6:**
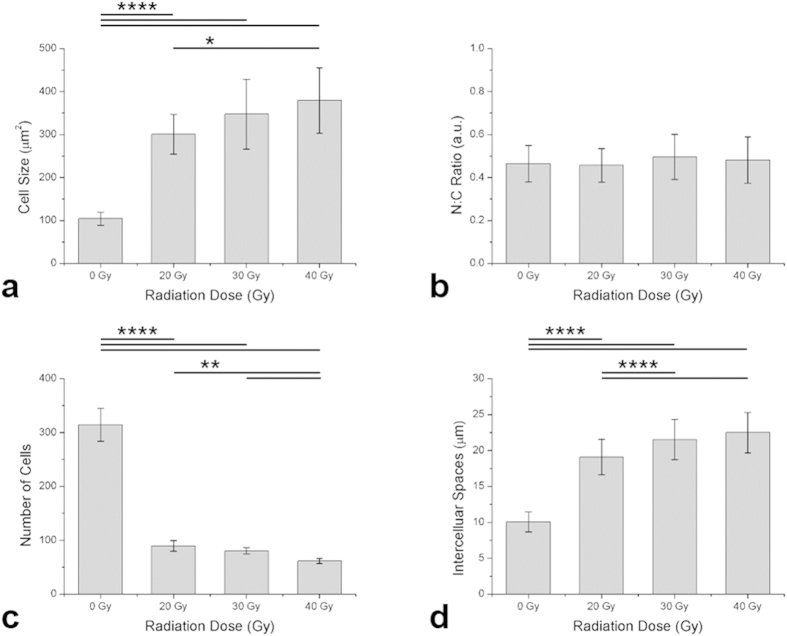
Quantitative analysis of TP cell images in the stratum basale. (**a**) cell size (n = 23), (**b**) NC ratio (n = 23), (**c**) cell density (n = 6), (**d**) intercellular spaces (n = 170). Error bar indicates standard deviation. The mean values and respective standard deviations are shown in bar graph. Statistical significance were presented by number asterisks depending on the p-value: ****p < 0.0001, **p < 0.01, *p < 0.05.

Cellular changes found TP images of the stratum basale on day 6 after irradiation in Fig. 1 were analyzed quantitatively, and the results are shown in Fig. 6. The individual cell size, nucleus-cytoplasm (N:C) ratio, number of cells, and intercellular space are shown in Fig. 6(a–d), respectively. After irradiation, the cell size and intercellular space increased and the number of cells decreased. The cell size between the 0 Gy (control) and irradiated groups show approximately 3 to 4-fold differences (p < 0.0001) in Fig. 6(a). There is significant difference between radiation groups: 20 Gy and 40 Gy (p < 0.05). In case of N:C ratio in Fig. 6(b), no statistical significance is found between the control and the irradiated groups, despite the difference in the cell size. The number of cells shows approximately 2 to 3-fold differences between of the control and irradiated groups (p < 0.0001) in Fig. 6(c). There are significant differences among radiation groups: 20 Gy and 40 Gy, 30 Gy and 40 Gy (p < 0.01). The intercellular space shows approximately 2-fold difference between of the control and irradiated groups of 20 Gy, 30 Gy, and 40 Gy (p < 0.0001) in Fig. 6(d). There are significant differences among radiation groups: 20 Gy and 30 Gy, 20 Gy and 40 Gy (p < 0.0001).

## Discussion

In this study, early-stage changes of the IR-induced skin injuries were characterized at the cellular level *in vivo* in a mouse model using TPM. Cellular structures in the epidermis and sebaceous gland were visualized in good contrast based on autofluorescence, and various cellular changes were observed in the early stage of IR injury. The most noticeable changes were those in cell morphology and distribution. Cells in the epidermis became larger and irregular due to IR injury. Cells with multiple nuclei were found, indicating mitotic abnormality. Intercellular space, which was regular and relatively narrow in the control skin, became wide and irregular. Cell density decreased compared to that of the control skin. Sebaceous glands in the dermis became smaller and disappeared, probably due to cell damage. Such microscopic cellular changes seemed to precede the macroscopic symptoms, since these cells in the skin are highly proliferative and sensitive to IR damage. These cellular changes found by TP imaging were consistent with white light imaging and histology results. White light images showed hair loss on day 6 after irradiation, which was consistent with TPM results of the sebaceous gland. Histological images showed thickened epithelium with irregular cell arrangement, loss of the hair follicle and sebaceous glands, and various changes in the dermis.

Optical imaging techniques have been developed and applied as the diagnostic method of IR skin injury to overcome limitations of previous methods. Optical techniques can non-invasively screen the injury region by detecting various changes in the skin such as vasculature and skin cells *in vivo*. Optical imaging techniques such as HSI, DOS, and LSI detect changes in blood vessels due to endothelial damage, and RCM treats changes in epithelium and inflammation at the cellular level. In this study, TPM was used to detect cellular changes in the epithelium, similarly to RCM. TPM visualized cell morphology including cell nucleus and cell distribution based on autofluorescence in good contrast, and could detect various cellular changes due to IR irradiation in more detail than RCM. Although TPM is very sensitive to cellular changes in the skin epithelium and sebaceous glands, TPM has several limitations in the field of view (FOV) and imaging speed, etc. TPM has a small FOV of less than 1 mm squared typically due to using an objective lens for high resolution imaging. TPM has a slow imaging speed of approximately a few seconds per frame and minutes per volume, due to weak autofluorescence signals. The small FOV and slow imaging speed makes TPM difficult to screen large skin regions, and sampled screening may be needed at the moment. However, there are ways to increase the imaging speed: (1) to use high-speed TPM utilizing multiple excitation foci^28^ or polygonal mirror scanner^29^, (2) to use an extrinsic contrast agent which is safe for human use, (3) to optimize laser parameters. Therefore, TPM may have good potentials for detecting early-stage changes of IR skin injury, and further development is needed in order for it to be utilized as a diagnostic method.

Besides the presented parameters used for the characterization of IR-induced skin injury, there still remain several functional and morphological changes that can be considered. Based on the autofluorescence intensity and fluorescence lifetime, the metabolic activity can be measured, which may aid differentiation of healthy and damaged cells in the skin^30^. From the histology results, the exposure to IR causes increase in immune response which may act as a parameter for differentiation of the healthy and irradiated skin. Dermatological approaches such as measurement of erythema index and pigmentation can be alternative methods for characterization^31^. Skin tissue assessment by using optical coherence tomography (OCT) could provide both structural and vascular information which may be used for early stages diagnosis.

## Materials and Methods

### Mouse model and irradiation

The experimental procedures were approved by Institutional Animal Care and Use Committee (IACUC, approval number KIRAMS2015-0004) of Korea Institute of Radiobiology & Medical Sciences (KIRAMS). All experiments were carried out in accordance with the approved guidelines and regulations. Sixteen 6-week-old male mice SKH1-HrHr hairless mice (Jackson Laboratory) were used in this study. The mice were kept under controlled conditions at POSTECH Biotech Centre (PBC), with constant temperature (23 ± 1 °C) and photoperiod (12 hours of light, 12 hours of dark). The mice were allowed free access to regular chow and 3-stage filtered water. In total, 16 mice were divided into 4 individual groups: 0 Gy (control) and irradiated groups of 20 Gy, 30 Gy, and 40 Gy (n = 4).

Mice were irradiated with different doses using a biological irradiator (XRAD-320, PRECISION X-RAY, Softex, Korea). For hind limb irradiation, animals were anesthetized by intraperitoneal injection with tiletamine-zolazepam (80 mg/kg, Zoletil®; Virbac Korea, Seoul, Korea) plus xylazine hydrochloride (10 mg/kg, Rompun®; Bayer Korea, Seoul, Korea), and positioned on the bed. The right hind limbs of the mice were longitudinally placed for IR exposure. The mice were irradiated with single exposure to X-ray at a dose rate of 2 Gy/min in a line area of 17 cm * 1 cm in total. The reference dose rate was established with an UNIDOSE®E universal dosimeter (PTW, Freiburg, Germany) in realistic irradiation conditions in air on the animal plate.

### Two-photon microscopy (TPM)

The right hind limbs of the mouse model were imaged using a commercial two-photon microscope (TCS SP5 II MP, Leica) with Ti-Sapphire laser (Chameleon Vision II, Coherent) with specifications of 140 fs pulse width and 80 MHz repetition rate. The excitation was tuneable ranging from 680 nm to 1080 nm, and was set to 740 nm for efficient excitation of intrinsic fluorophores including NADH. TP imaging was performed using a 25× objective lens (HCX IRAPO L25X, NA 0.95 W, Leica) with depth-wise increment of 1.5 μm. The emission signals from skin tissue were spectrally resolved to 4 channels of 457/50, 525/50, 585/40, and 650/50 by using a set of dichroic mirrors and band-pass emission filters and four non-descanned detectors (NDD) filter. The acquired images were presented in pseudocolor of green (457/50), yellow (525/50), and red (585/40). The size of images were 512 × 512 pixels covering a field of view of 620 μm × 620 μm or 207 μm × 207 μm. The imaging speed was 0.52 frames per second including 3 line averaging and 2 frame accumulation.

The TP skin imaging of the right hind limb of the mouse model was performed daily for 6 days. During the TP imaging, the body temperature of mice were maintained at 37 °C using temperature controlled heating plate (Chamlide TR, Live Cell Instrument) set on the microscope stage. The mice were anesthetized using a face mask administering gas mixture of 1.5%/vol isoflurane (Terrel™, Piramal) and medical grade oxygen.

To minimize the breathing motion during TP imaging, the hind limb was gently stretched and held using a custom-made hind limb holder and surgical tape. The hind limb holder was made of acryl and consisted of two parts: a base plate and a thin cover plate. The rectangular shaped base plate had four support fixtures consisting of M4 bolts and nuts at each corner. These fixtures were used to hold the mouse hind limb together with the cover plate. The cover plate had a square hole similar to the size of the microscope cover slip, where a cover glass was attached to the square hole for TP imaging. The attached cover glass gently pressed the mouse hind limb during imaging.

### White light images

Prior to the imaging, mice were anesthetised by intraperitoneal injection of tiletamine-zolazepam (80 mg/kg, Zoletil®; Virbac Korea, Seoul, Korea) and xylazine hydrochloride (10 mg/kg, Rompun®; Bayer Korea, Seoul, Korea), and were gently positioned on surgical pads. White light images of mouse right hind limb were acquired using colour complementary metal-oxide semiconductor (CMOS) camera in room light environment. The white light images were acquired equidistant from the sample surface throughout the experiment.

### Skin tissue histology

Histology analysis of IR-induced skin injuries were performed on day 6 of the experiment. Animals were anesthetized, euthanized, and necropsied. The skin of hind limbs were excised and fixed with 4% paraformaldehyde solution for 24 h. After fixation, the skin tissues were embedded in paraffin wax and were sectioned with a thickness of 4 μm. The sections were stained with hematoxylin and eosin (H&E) to assess microscopic findings.

### TPM image analysis

The acquired TP images were post-processed to increase the contrast and visibility and was used for quantitative analysis. The post-image processing and quantitative analysis were performed using provided software Leica Application Suite software (LAS AF Lite, Leica) and MATLAB (Matlab 2015a, Mathworks). The quantitative analysis of cell size, NC ratio, cell density, and intercellular space were calculated from the TP images of the stratum basale layer of epidermis acquired at day 6 of the experiment. Calculations for cell size and intercellular spaces were performed by using the total number of significant pixels for each parameter and the actual pixel size. For analysis of cell size, the area of cell including cytoplasm and nucleus were manually segmented following their borderline. From the segmented cell, the nucleus, which is depicted with negative contrast, is manually segmented to finally differentiate individual sizes of cytoplasm and nucleus. The sizes of segmented nucleus and cytoplasm were calculated based on the number of pixels within the area. For assessment of cell size, entire cell including cell nucleus and cell cytoplasm were used. The NC ratio were calculated using the area ratio of the nucleus and cytoplasm. In the presence of mitotic catastrophe, all the micronuclei were considered for measurement. The calculations for cell density were performed by manual counting of visible cells in the stratum basale. The intercellular spaces were calculated measuring the spaces between the manually selected centre points of the neighbouring cells. The closest intercellular spaces were selected for comparison.

### Statistical analysis

Statistical analysis (statistical significance) was performed for quantitative analysis by using GraphPad Prism 6 (GraphPad Software Inc., La Jolla, CA). Results are expressed as mean ± standard deviation (SD). Unpaired student’s t-test were used for intergroup comparisons. All statistical test were performed by two-tailed test and p-values  < 0.05 were considered as criterion for statistical significance. Statistical significance were presented by number asterisks depending on the p-value; ****p < 0.0001, **p < 0.01, *p < 0.05.

## Additional Information

**How to cite this article**: Hyuk Jang, W. *et al.* *In vivo* characterization of early-stage radiation skin injury in a mouse model by two-photon microscopy. *Sci. Rep.* **6**, 19216; doi: 10.1038/srep19216 (2016).

## Supplementary Material

Supplementary Video 1

Supplementary Video 2

Supplementary Video 3

Supplementary Video 4

Supplementary Information
