# Supplementary material for: In vivo characterization of early-stage radiation skin injury in a mouse model by two-photon microscopy
Source: Sci Rep. 2016 Jan 12;6:19216. doi: 10.1038/srep19216 (PMC4709756; doi:10.1038/srep19216)
Supplement: Supplementary Information [file srep19216-s5.doc]

**Supplementary Information**

In vivo characterization of early-stage radiation skin injury in a mouse model by two-photon microscopy

Won Hyuk Jang^1, +^, Sehwan Shim^2, +^, Taejun Wang^1^, Yeoreum Yoon^3^, Won-Suk Jang^4^, Jae Kyung Myung^, 2, 4, 5^, Sunhoo Park^2, 4, 5 *^, Ki Hean Kim^1, 3, *^

**Affiliations:**

^1^Divison of Integrative Biosciences & Biotechnology, Pohang University of Science and Technology, 77 Cheongam-Ro, Nam-gu, Pohang, Gyeongbuk 37673, Rep. of Korea

^2^National Radiation Emergency Medical Centre, Korea Cancer Centre Hospital, Korea Institute of Radiological & Medical Sciences (KIRAMS), 75 Nowon-ro, Nowon-gu, Seoul 01812, Rep. of Korea

^3^Department of Mechanical Engineering, Pohang University of Science and Technology, 77 Cheongam-Ro, Nam-gu, Pohang, Gyeongbuk 37673, Rep. of Korea

^4^Laboratory of Experimental Pathology, Korea Cancer Centre Hospital, Korea Institute of Radiological & Medical Sciences (KIRAMS), 75 Nowon-ro, Nowon-gu, Seoul 01812, Rep. of Korea

^5^Department of Pathology, Korea Cancer Centre Hospital, Korea Institute of Radiological & Medical Sciences (KIRAMS), 75 Nowon-ro, Nowon-gu, Seoul 01812, Rep. of Korea

*corresponding author: [sunhoo@kcch.re.kr](mailto:sunhoo@kcch.re.kr), [kiheankim@postech.ac.kr](mailto:kiheankim@postech.ac.kr)

+these authors contributed equally to this work

**Contents**

**Supplementary Video legends**

Supplementary Video 1: Representative three-dimensional volume scan of two-photon microscopy images of 0 Gy mouse group acquired on day 6 after irradiation. The image size is 512 x 512 pixels covering a field of view of 207 μm x 207 μm and was depth-wised scanned in increment of 1.5 μm. The video playback is at 5 frames per seconds. (See main Fig. 1a)

Supplementary Video 2: Representative three-dimensional volume scan of two-photon microscopy images of 20 Gy mouse group acquired on day 6 after irradiation. The image size is 512 x 512 pixels covering a field of view of 207 μm x 207 μm and was depth-wised scanned in increment of 1.5 μm. The video playback is at 5 frames per seconds. (See main Fig. 1b)

Supplementary Video 3: Representative three-dimensional volume scan of two-photon microscopy images of 30 Gy mouse group acquired on day 6 after irradiation. The image size is 512 x 512 pixels covering a field of view of 207 μm x 207 μm and was depth-wised scanned in increment of 1.5 μm. The video playback is at 5 frames per seconds. (See main Fig. 1c)

Supplementary Video 4: Representative three-dimensional volume scan of two-photon microscopy images of 40 Gy mouse group acquired on day 6 after irradiation. The image size is 512 x 512 pixels covering a field of view of 207 μm x 207 μm and was depth-wised scanned in increment of 1.5 μm. The video playback is at 5 frames per seconds. (See main Fig. 1d)
